# Vestibular paroxysmia entails vestibular nerve function, microstructure and endolymphatic space changes linked to root-entry zone neurovascular compression

**DOI:** 10.1007/s00415-022-11399-y

**Published:** 2022-10-18

**Authors:** Emilie Kierig, Johannes Gerb, Rainer Boegle, Birgit Ertl-Wagner, Marianne Dieterich, Valerie Kirsch

**Affiliations:** 1 Department of Neurology, University Hospital, Ludwig-Maximilians-Universität, Fraunhoferstr. 20, 82152 Munich, Germany; 2 German Center for Vertigo and Balance Disorders-IFB, University Hospital, Ludwig-Maximilians-Universität, Munich, Germany; 3 Graduate School of Systemic Neuroscience (GSN), Ludwig-Maximilians-Universität, Munich, Germany; 4 Department of Radiology, The Hospital for Sick Children, University of Toronto, Toronto, Canada; 5 Department of Radiology, University Hospital, Ludwig-Maximilians-Universität, Munich, Germany; 6 Munich Cluster for Systems Neurology (SyNergy), Munich, Germany

**Keywords:** Vestibular paroxysmia, Endolymphatic hydrops, Endolymphatic space, MRI, Inner ear, Gadolinium-based contrast agent, Intravenous, Vestibular nerve, Root-entry zone, DTI, Diffusion imaging

## Abstract

Combining magnetic resonance imaging (MRI) sequences that permit the determination of vestibular nerve angulation (NA = change of nerve caliber or direction), structural nerve integrity via diffusion tensor imaging (DTI), and exclusion of endolymphatic hydrops (ELH) via delayed gadolinium-enhanced MRI of the inner ear (*i*MRI) could increase the diagnostic accuracy in patients with vestibular paroxysmia (VP). Thirty-six participants were examined, 18 with VP (52.6 ± 18.1 years) and 18 age-matched with normal vestibulocochlear testing (NP 50.3 ± 16.5 years). This study investigated whether (i)* NA, *(ii) DTI changes, or (iii) ELH occur in VP, and (iv) to what extent said parameters relate. Methods included vestibulocochlear testing and MRI data analyses for neurovascular compression (NVC) and NA verification, DTI and ELS quantification. As a result, (i) NA increased NVC specificity. (ii) DTI structural integrity was reduced on the side affected by VP (*p* < 0.05). (iii) 61.1% VP showed mild ELH and higher asymmetry indices than NP (*p* > 0.05). (iv) “Disease duration” and “total number of attacks” correlated with the decreased structural integrity of the affected nerve in DTI (*p* < 0.001). NVC distance within the nerve’s root-entry zone correlated with nerve function (Roh = 0.72, *p* < 0.001), nerve integrity loss (Roh = − 0.638, *p* < 0.001), and ELS volume (Roh = − 0.604, *p* < 0.001) in VP. In conclusion, this study is the first to link eighth cranial nerve function, microstructure, and ELS changes in VP to clinical features and increased vulnerability of NVC in the root-entry zone. Combined MRI with NVC or NA verification, DTI and ELS quantification increased the diagnostic accuracy at group-level but did not suffice to diagnose VP on a single-subject level due to individual variability and lack of diagnostic specificity.

## Introduction

Vestibular paroxysmia (VP) is a rare cause of short episodic vertigo with or without auditory and vegetative symptoms [1]. The assumed mechanism is ephaptic discharges induced by demyelination with succeeding hyperexcitability through neurovascular compression (NVC) in the root-entry and transition zone of the eighth cranial nerve [2–4]. Diagnostic criteria for VP, as defined by the Classification Committee of the Bárány Society, are mostly based on patient history [5]. However, due to symptom variability caused partly by an affection of different audiovestibular parts of the eighth cranial nerve, diagnosis is only considered definitive when responsive to carbamazepine or oxcarbazepine.

Clinical and neurophysiological parameters vary in VP. Patients can exhibit signs of mild to moderate unilateral nerve failure [6, 7], excitation [8, 9], both [10, 11], or no dysfunction [12] in audiovestibular testing during attack-free intervals. Therefore, a combination of neurophysiological and imaging techniques is recommended to determine the affected side and the degree of damage associated with VP [6]. However, magnetic resonance imaging (MRI) findings of vascular compression are not VP-specific or predictive for the affected side because they are also observed in about 30% of healthy asymptomatic subjects [6, 10]. Moreover, 7 Tesla T1 structural MRI detected no abnormalities within the VP vestibular nerve in six patients underlining that VP symptoms are not a sign of nerve hypofunction [13].

The question was whether an MR imaging approach tailored to VP could improve the overall predictive diagnostic value in VP. Therefore, the combination of three sequences permits the determination of vestibular nerve angulation, structural nerve integrity via diffusion tensor imaging (DTI), and non-invasive quantification of the endolymphatic space (ELS) by delayed gadolinium-enhanced MRI of the inner ear (iMRI) were used. Nerve angulation/distortion (NA) was proposed to be a more specific imaging feature than NVC [14]. In addition, DTI revealed significantly lower anisotropy and higher apparent diffusion coefficient in the affected trigeminal root in trigeminal neuralgia than healthy controls [15]. Furthermore, iMRI is developing into a standard clinical tool to investigate a possible endolymphatic hydrops (ELH) as a cause of episodic vertigo, such as in Ménière’s disease [16, 17] and vestibular migraine [18, 19]. Against this background, the study investigated the following aspects:

(i) Can nerve angulation/distortion improve the NVC informative value in VP?
(ii) Are there measurable structural differences in the affected eighth cranial nerve in VP?
(iii) Does ELH play a role in VP?
(iv) To what extent are clinical, diagnostic, and MRI VP parameters related?

## Materials and methods

### Setting and institutional review board approval

All data were acquired at the Interdisciplinary German Center for Vertigo and Balance Disorders (DSGZ) and the Neurology Department of the Munich University Hospital (LMU) between 2018 and 2020. Institutional Review Board approval was obtained before the initiation of the study (no. 641-15). Furthermore, all participants provided informed oral and written consent in accordance with the Declaration of Helsinki before inclusion in the study.

### Study population

Thirty-six consecutive participants, 18 patients with vestibular paroxysmia (VP) and 18 age-matched participants with normal vestibulocochlear testing (NP), underwent MR imaging with sequences that permit determination of vestibular nerve angulation, structural nerve integrity via diffusion tensor imaging (DTI), and delayed intravenous gadolinium-enhanced magnetic resonance imaging (iMRI) for exclusion or verification of ELH. Diagnosis of VP was based on the Classification Committee of the Bárány Society 2016 [5]. Therefore, MRI results were excluded from the diagnostic classification. NP were inpatients of the Neurology Department without symptoms or underlying pathologies of the vestibulocochlear system that underwent MRI with a contrast agent as part of their diagnostic workup and agreed to undergo additional iMRI sequences after 4 h. Ethical considerations did not allow us to include healthy volunteers without a medical indication for an iMRI with a contrast agent (see limitations for more information). NP underwent audiovestibular testing to confirm the soundness of their peripheral inner ear end-organs. The reasons for their admission to the clinic included movement disorders (*n* = 4), epilepsy (*n* = 3), optic neuritis (*n* = 2), polyneuropathy (*n* = 2), headache (*n* = 2), idiopathic facial nerve palsy (*n* = 1), viral meningitis (*n* = 1), subdural hematoma (*n* = 1), spinal inflammatory lesion (*n* = 1), and decompensated esophoria (*n* = 1). The laterality quotient for right-handedness was assessed with the ten-item inventory of the Edinburgh test [20]. Inclusion criteria were age between 18 and 85 years (VP, NP) and normal audiovestibular testing to confirm the soundness of their peripheral end-organs and the central vestibular system (NP, see “Measurement of the auditory, semicircular canal and otolith functions”). Exclusion criteria for NP were current cochlear or vestibular disorders, a positive history of vertigo-, balance-, or hearing disorders, as well as any MR-related contraindications [21], poor image quality, or missing MR sequences for NP and VP.

### Measurement of the auditory, semicircular canal and otolith functions

Diagnostic workup included a thorough neurological workup (e.g., history-taking, clinical examination), neuro-orthoptic assessment [e.g., Frenzel glasses, fundus photography and adjustments of the subjective visual vertical (SVV)], video-oculography (VOG) during caloric and head impulse testing (HIT), as well as ocular and cervical vestibular evoked myogenic potentials (o/cVEMPs), and pure tone audiometry (PTA).

A tilt of the SVV is a sensitive sign of an acute graviceptive vestibular tone imbalance. SVV was assessed with the subject sitting upright in front of a half-spherical dome with the head fixed on a chin rest [22]. A mean deviation of > 2.5° from the true vertical was considered a pathological tilt of SVV [22].

The impairment of the vestibulo-ocular reflex (VOR) in higher frequencies was measured by HIT [23] using high-frame-rate VOG with EyeSeeCam ([24], EyeSeeTech, Munich, Germany). A median gain during head impulses < 0.6 (eye velocity in °/s divided by head velocity in °/s) was considered a pathological VOR [25]. Furthermore, semicircular canal responsiveness in lower frequencies was performed for both ears with 30° cold and 44° warm water. Vestibular paresis was defined as > 25% asymmetry between the right- and left-sided responses [26] or the sum of the maximal peak velocities of the slow phase caloric-induced nystagmus for stimulation with warm and cold water on each side < 25°/sec [27]. The caloric asymmetry index (AI_C_) was calculated based on the slow-phase velocity of the caloric nystagmus:${AI}_{C }[\%] =\frac{({R}_{33^\circ }+{R}_{44^\circ })-({L}_{33^\circ }+{L}_{44^\circ })}{({R}_{33^\circ }+{R}_{44^\circ })+({L}_{33^\circ }+{L}_{44^\circ })}\times 100.$

Vestibular evoked myogenic potentials (VEMPs) are short-latency, mainly otolith-driven vestibular reflexes elicited by air-conducted sound (ACS), bone-conducted vibration, or galvanic vestibular stimulation and recorded from the contralateral inferior oblique eye muscle (ocular or oVEMPs) or the ipsilateral sternocleidomastoid muscle (cervical or cVEMPs). VEMPs were recorded with the Eclipse platform (Interacoustics, Middelfart, Denmark) as described previously [28, 29]. VEMP responses that were discernible from background noise were included in the analysis. Furthermore, only the asymmetry index (AI_O/CV_) of VEMP amplitudes and latencies were analyzed to avoid bias by examiners [30]. Amplitude side difference ≥ 50% was considered pathological.

For cochlear function and acoustic processing, acoustically evoked potentials (AEP) and pure-tone audiometry (PTA) by air conduction at 250 Hz–8.0 kHz were performed. PTA was based on both ears' four-tone average (arithmetic mean) of the thresholds at 0.5, 1, 2, and 3 kHz. Hearing loss was defined as PTA > 25 dB [31]. In all tests, the contralateral ear was masked by adequate noise. All audiometric equipment is regularly recalibrated (every 6 months) according to the local university equipment standard.

### Nomenclature

In the following, “ipsilateral” refers to the clinically leading side (or affected side) and “contralateral” to the opposite side (or non-affected side). The definition of the “clinically leading side (or affected side)” was based on clinical and neurophysiological findings. Side–defining signs were clear patterns of unilateral loss of audiovestibular function [10]. In the case of patients presenting without a leading clinical side, a pseudorandom number generator [“Mersenne Twister” algorithm [31], uniform distribution] was used to generate a random number between 1 (= minimum value) and 9 (= maximum value). Even numbers meant “left side = ipsilateral side,” and uneven numbers indicated “right = ipsilateral side.” “Vertigo” pertains to attacks of spontaneous spinning or non-spinning vertigo with a duration of less than 1 min, stereotyped phenomenology in a particular patient that fits the criteria of VP [5], and is not better accounted for by another diagnosis.

### MR imaging approach tailored to VP

**Fig. 1:**
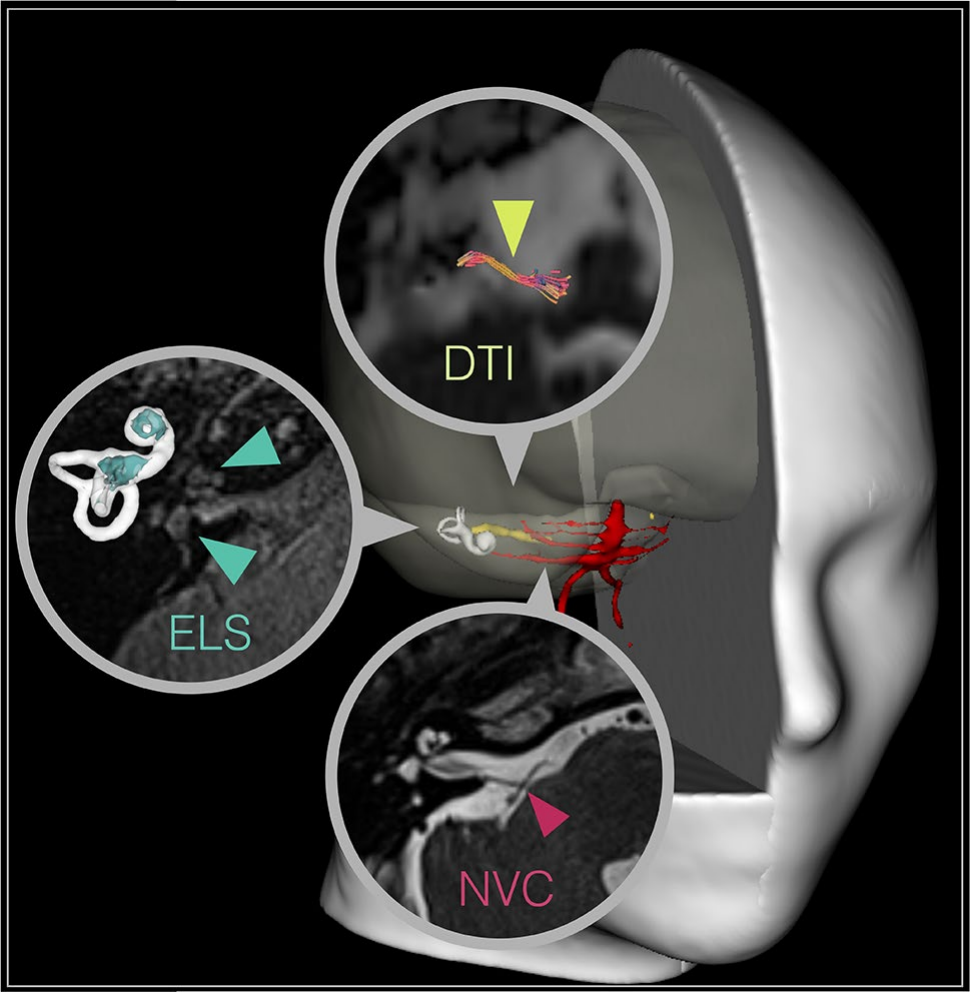
MR imaging approach tailored to VP. This study investigated the vestibular nerve with a MR imaging approach tailored to vestibular paroxysmia (VP). A 3D rendered T1-image visualizes the spatial relationship between overlaid blood vessels (red), the eighth cranial nerve (8 CN, yellow), and the vestibulocochlear end organ (white shell). These key structures are further shown magnified in a circle within a transversal slice of the cerebellopontine angle in the respective raw data with color-coded arrowheads pointing towards them. Particular emphasis was put on the site of neurovascular compression (NVC, behind red arrowhead in the lower circle), and the DTI tractography quantification of the cisternal segment of the eighth cranial nerve (behind yellow arrowhead in the upper circle) between the point of entry (PE: point of entry for afferent fibers, point of exit for efferent fibers into/out of the brainstem) and the internal auditory canal (IAC). In addition, the study focused on the volumetric quantification of the endolymphatic space (ELS, behind the turquoise arrowheads in the middle circle) within the membranous and bony labyrinth of the inner ear. The upper arrowhead points toward the cochlea, and the lower arrowhead points towards the vestibulum. The corresponding volumetric visualization of these structures (ELS in turquopoise) is depicted on the left, next to these structures. Visualization tools were a combination of 3D Slicer (https://www.slicer.org/, version 4.11 [36]) and DSI Studio (http://dsi-studio.labsolver.org/, version 2021-02-12).

MR imaging in this study used combined sequences to investigate the peripheral vestibular system in VP (see Fig. 1). Particular emphasis was put on the presence of neurovascular compression (NVC) and structural DTI tractography quantification along the intracisternal course of the eighth cranial nerve. Another focus included the volumetric quantification of the endolymphatic space (ELS) within the membranous and bony labyrinth of the audiovestibular end-organ.

#### Data acquisition

Four hours after intravenous injection of a standard dose (0.1 mmol/kg body weight) of gadobutrol (Gadovist^®^, Bayer, Leverkusen, Germany), MR imaging (MRI) data were acquired in a whole-body 3 Tesla MRI scanner (Magnetom Skyra, Siemens Healthcare, Erlangen, Germany) with a 20-channel head coil.

A high-resolution, strongly T2-weighted, spin-echo 3D-SPACE (three-dimensional **s**ampling **p**erfection with **a**pplication-optimized **c**ontrasts using different flip angle **e**volutions) sequence delineated the total inner ear fluid space from the surrounding bone. A T2-weighted 3D-FLAIR (three-dimensional **fl**uid-**a**ttenuated **i**nversion **r**ecovery) sequence was used to differentiate endolymph from perilymph within the total fluid space (TFS). The 3D-FLAIR sequence had the following parameters: TR 6000 ms; TE 134 ms; TI 2240 ms; FA 180°; FOV 160 × 160 mm^2^; 36 slices; base resolution 320; averages 1; slice thickness 0.5 mm. The 3D-SPACE sequence had the following parameters: TR 1000 ms; TE 133 ms; FA 100°; FOV 192 × 192 mm^2^; 56 slices; base resolution 384; averages 4; slice thickness of 0.5. According to a previously reported method [32], ELS was observed on the 3D-FLAIR images as enlarged negative-signal spaces inside the labyrinth.

T1 and DTI structural sequences were used to localize and quantify the vestibular nerve. T1-weighted magnetization-prepared rapid gradient echo (MP-RAGE) sequences had the following parameters: FOV 256 mm; isotropic spatial resolution of 1.0 × 1.0 × 1.0 mm^3^; TE 2.07 ms; TR = 1900 ms; number of slices 160. Diffusion tensor imaging (DTI) sequences were measured with the following parameters: 64 directions; 2.0 × 2.0 × 2.0 mm^3^ isotropic voxels; TE = 95 ms; TR = 9600 ms, bipolar diffusion scheme; b values = 0 and 1000 s/mm^2^.

#### Data analyses

##### Neurovascular compression and nerve angulation of the eighth cranial nerve

The vestibulocochlear (eighth cranial) nerve was examined using a double oblique reformat of the 3D-SPACE data, with the sagittal plane set in a modified glabellomeatal line and the coronal plane set in line with the vestibulocochlear nerve as suggested by [14]. An experienced head and neck radiologist (BEW) and two neurologists (EK, VK), blinded to the clinical diagnosis, assessed the data. If discrepancies arose, a consensus was reached by discussion. Neurovascular compression (NVC) was defined as a lack of a detectable layer of cerebrospinal fluid between the eighth cranial nerve and any surrounding blood vessel, as proposed by reference [10]. All identified vessels were followed to their origin and classified anatomically. It was classified as an artery (vein) if retraceable to a major arterial (venous) vessel. NVC between the point of entry (PE: point of entry for afferent fibers, point of exit for efferent fibers into/out of the brainstem) and the beginning of the internal auditory canal (IAC) were called ‘cisternal,’ NVC within the IAC ‘meatal.’ Nerve angulation (NA) was defined as a change in direction or caliber of the vestibulocochlear nerve at the point of contact, as proposed previously [14]. VP patients had been diagnosed before MRI acquisition based on the clinical diagnostic criteria.

##### Quantitative structural DTI assessment of the eighth cranial nerve

DTI preprocessing included noise correction using MRTRIX3 [33–35]. T1-MPRAGE volume was manually cropped using 3D-SLICER version 4.10.2 [36]. Automated brain extraction was performed with FSL BET [37]. An artificial b0 volume was created using SynB0 [38] and used for geometric and susceptibility artifact correction using FSL Topup [39] and Eddy [40]. After bias field correction using ANTS [41], a reference b0 was extracted from the geometrically corrected **d**iffusion-**w**eighted **i**maging (DWI). The preprocessed DWI was interpolated to a voxel size of 1 mm^3^ using MRTRIX3. A two-step-registration with 12 degrees of freedom was performed using the BRAINSFIT module included in 3D-Slicer, with (j) coregistration of the T1-MPRAGE (moving image) and the reference b0 (fixed image) and.”j) coregistration of the high-resolution 3D-SPACE (moving image) and the registered T1-MPRAGE* (fixed image). Finally, a fusion volume of b0 and registered 3D-SPACE was created using MRTRIX3.

DTI tractography and tract-specific analyses were performed using DSI-Studio ([42], http://dsi-studio.labsolver.org/). First, an automatic quality control routine checked the b-table to ensure its accuracy [38]. Next, the diffusion tensor was calculated. A deterministic fiber tracking algorithm [43] was used with augmented tracking strategies [44] to improve reproducibility. A spherical seed region was placed at the IAC on the b0-SPACE fusion volume; a spherical ending region was placed at the root-entry zone of the eighth cranial nerve at the pontocerebellar angle. The threshold settings were as follows: anisotropy threshold: 0.5–0.7 Otsu’s threshold, change threshold: 20%, angular threshold: 15–90 degrees, step size: 0.5–1.5 voxels. Tracks shorter than 15 mm or longer than 150 mm were discarded. After 500 tracts were calculated, fiber tracking was automatically terminated. Based on this first approximation, the vestibulocochlear nerve was selected based on the following criteria: (k) anatomically plausible direction, (kk) brainstem entry at the pontocerebellar angle, and (kkk) visual correlation with the course of the nerve in the b0-SPACE fusion volume. After identifying the nerve, the tracts were manually adjusted to remove unwanted fibers inside the brainstem, e.g., the vestibulospinal tract. The generated tracts for the eighth cranial nerves on both sides were stretched to an equal length, allowing analysis of DTI parameters along the course of the tract. A regression kernel size of 2.0 was used.

DTI indices over fractional anisotropy (FA), mean diffusivity (MD), axial diffusivity (AD), and radial diffusivity (RD) were investigated in the following three ways: *(*l*) Average tensor map approach:* The average DTI indices of the entire vestibular nerve (VN) were used as a surrogate marker for the overall VN integrity. *(*ll*) Localized tensor map approach:* Fiber tracts were assessed in 100 1% subsegments. The entire length (between PE and IAC) of each subject’s VN fiber was 100%. In combination with the affected side, the calculation of the NVC subsegment [%] = $\frac{\mathrm{PE to NVC }[\mathrm{mm}]}{\mathrm{PE to IAC }[\mathrm{mm}]}\times 100$ enabled a localized NVC-specific assessment of VN. *(*lll*) Successive differences tensor map approach*: The 100 generated 1% subsegments were transformed into statistical summary parameters (SSP) to reduce the statistical testing and extract relevant features. SSP assessing the “overall” effects of VP vs. NP included the “mean”, “median”, “standard deviation”, and “interquartile range” across all 100 generated 1% steps, i.e., treating each part of the nerve similarly to every other part, as though each segment were a random manifestation of one overall effect. Furthermore, the deviations along the nerve were assessed via statistics of successive differences of first (1st) and second (2nd) order. Successive differences of 1st order are the differences between every two adjacent values of the 100 generated 1% steps divided by the first value to get a percentage. The 2nd order is the repetition of the process using the generated 99 differences of the 1st order step. The statistical parameters then calculated were *the mean of the squared differences* (MSSD) and *the mean of the absolute difference*s (MASD). Note that the squared differences allow a more substantial weighting of large deviations even if few, while the absolute differences do not weigh the larger deviations more strongly than many small ones. Therefore, *the mean squared successive differences* (MSSD: 1st order; MS2SD: 2nd order), and *the mean absolute successive difference*s (MASD: 1st order; MA2SD: 2nd order) were calculated as a measure of the relative influence of larger deviations. Due to the use of successive differences, SSPs treat local parts of effectively 2 (1st order) and 4 (2nd order) segments of the nerve as though they are realizations of a random process related to successive local deteriorations of the nerve. Differential entropy [45] was calculated using the 15, 27, and 50 nearest neighbors. SSP allows us to assess the “disorderedness” of the whole nerve and the successive differences. Each of these parameter classes was grouped into collections, such as “central tendencies” (mean and median), “dispersions” (standard deviation and interquartile range), “successive differences 1st order” (MSSD, MASD, and MSSD/MASD), “successive differences 2nd order” (MS2SD, MA2SD, and MS2SD/MA2SD fraction), “raw entropy” and “successive difference entropy''. SSP group differences were assessed using principal component analysis (PCA) using MATLAB’s built-in *pca.m* in the statistics and machine learning toolbox. SSP correlations to chosen clinical parameters (such as “disease duration”, “total number of attacks”, “the average length of the attack”, and “the number of attacks in the last three months”) were tested by projection into latent space (PLS, [46–48]).

##### Semi-quantitative visual grading and 3D-quantification of the endolymphatic space

Semi-quantitative (SQ) visual grading of the endolymphatic space (ELS) was performed independently by an experienced head and neck radiologist (BEW) and two neurologists (JG, VK) who were blinded to the clinical patient data. The ELS’s characterization in the vestibulum and cochlea was based on previously described criteria [49].

3D-quantification of the ELS consisted of three steps: first, segmentation of the total fluid space (TFS) was based on IE-Vnet [50], a recently proposed and pre-trained volumetric deep learning algorithm with V-net architecture that was deployed via the TOMAAT module [51] in a 3D–Slicer toolbox (version 4.11 [36]). Second, ELS and perilymphatic space (PS) were differentiated within the TFS using **Vo**lumetric **L**ocal **T**hresholding (VOLT; [52]) with ImageJ Fiji [53], the “Fuzzy and artificial neural networks image processing toolbox” [54], and the “MorphoLibJ Toolbox” [55]. The resulting 3D volume included classification into two different compartments (ELS and PS), examined at cutoff 6. Third, measurements were performed using the ‘Analyze Regions (3D)’ plugin of the “MorpholibJ Toolbox” [55]. The method is described in more detail in previous publications [52].

ELS symmetry between both inner ears was assessed using two parameters: Absolute ELS side difference, where $\Delta =|{\mathrm{ELS}}_{i}-{\mathrm{ELS}}_{c}|$, and ELS_*i*_ is the 3D-quantification of the ipsilateral ELS and *ELS*_*c*_ of the contralateral ELS in mm^3^. Normalized ELS side difference, with an asymmetry index, $\mathrm{AI} [\%]=\frac{({\mathrm{ELS}}_{i}-{\mathrm{ELS}}_{c})}{({\mathrm{ELS}}_{i} +{\mathrm{ELS}}_{c})}\times 100$, where the value is independent of the individual TFS.

### Statistics and validation parameters

Analyses were performed using the Statistical Package for Social Sciences software (SPSS, Inc, Chicago, IL, USA) or using self-written scripts in MATLAB, version 7.19.0 (R2019b), including the “Statistics and Machine Learning” toolbox provided with MATLAB (Natick, Massachusetts: The MathWorks Inc). Categorical values are reported as the number of cases that fit the category/number of patients in the examined group [%]; ordinal or scalar values are presented as (mean ± standard deviation).

#### NVC and ELS parameter

One-way analysis of variance (ANOVA) for multiple comparisons, which was post-hoc Bonferroni-corrected for multiple testing, was used for scalar (ELS volumetric quantification result, clinical diagnostic raw data) and ordinal (ELH semi-quantitative visual scoring result) values. Group differences were assessed between all VP vs. NP and definite VP vs. NP. Linear agreement between parameter pairs was calculated for each method separately using the two-sided Spearman’s correlation coefficient. Results were reported at a significance level of *p* < 0.05 and *p* < 0.001, corrected for multiple comparisons.

#### DTI parameter

DTI continuous data types were assessed for differences in “central tendency “(e.g., average or median) and “dispersion” (e.g., variance) between groups (VP vs. NP). The group differences were assessed using the non-parametric Wilcoxon rank-sum test for differences in medians between the groups and via the Brown–Forsythe test for group differences in variance of the two groups via the median absolute deviation. The collections named above (“central tendencies”, “dispersions”, “successive differences 1st order”, “successive differences 2nd order”, “raw entropy,” and “successive difference entropy”) were aggregated into single variables using principal component analysis (PCA) and choosing only the most significant principal component score as the representation of the collection for group difference assessment. The group differences were assessed by the non-parametric Wilcoxon rank-sum test for differences in “medians” between the groups and via the Brown–Forsythe test for group differences in variance of the two groups via the “median absolute deviation”. The correlation of the “collections’’ to disease parameters, such as “disease duration”, “number of attacks”, “average duration of attacks”, and “number of attacks in the three months prior to examination”, was assessed via projection to latent space PLS analog to previous work [47, 48, 56]. In addition, rank-correlations were assessed as the input matrix for the singular value decomposition, which contained scores with ranks correlated to disease parameters. To test these correlations, we use permutation testing as suggested in the previous work [48], using 1000 permutations of the disease parameter in question and counting each correlation more significant than the reference (unpermuted) correlation as a failure of the test, setting the p-value of the test as the number of failures divided by the total number of permutations, i.e., *p* < 0.001.

#### Interparametric correlations

Correlations between clinical and diagnostic features, NVC distances [mm], structural DTI quantifications, and ELS volumes were assessed with a PLS approach (analog to “Interparametric correlations”). Results were reported at correlation coefficient > 0.5 for all measures and significant in 1000 permutation tests, i.e., *p* < 0.001.

## Results

### Clinical syndrome and treatment in VP patients

Eighteen VP patients (6 females; aged 28–84 years, mean age 52.6 ± 18.1 years; 17 right-handed (RH), one left-handed (LH)] and 18 age-matched NP (13 females; aged 25–78 years, mean age 50.3 ± 16.5 years; 18 RH) were included in the study. VP patients could be further divided into ten definite (dVP, three females; aged 29–61 years, mean age 52.5 ± 8.8 years; 10 RH) and eight probable VP patients (pVP, four females; aged 49–77 years, mean age 63.6 ± 8.9 years; 7 RH, 1 LH). A detailed description of the VP groups’ symptomatology with accompanying complaints and signs, duration of attacks and disorder, and therapeutic success are given in Table 1.

**Table 1:** Clinical syndrome and treatment effects

	VP		
	All	Definite	Probable
Vertigo	55.6% Rotational 33.3% To-and-fro 11.1% Lightheadedness	60% Rotational 30% To-and-fro 10% Lightheadedness	50% Rotational 37.5% To-and-fro 12.5% Lightheadedness
Spontaneous onset	16/18 (88.9%)	8/10 (80%)	8/8 (100%)
Provocation of syndrome	44% No provoking factors 11% Ipsilateral head rotation 33% Bilateral head rotation 5% Head inclination 33% Head reclination	50% No provoking factors 20% Ipsilateral head rotation 20% Bilateral head rotation 10% Head inclination -	37.5% No provoking factors - 50% Bilateral head rotation - 12.5% Head reclination
Frequency of attacks	55.6% Several times/day 27.8% 2–3 times/week 11.1% 2–3 times/month 5.6% once/month	60% Several times/day 30% 2–3 times/week 10% 2–3 times/month -	50% Several times/day 25% 2–3 times/week 12.5% 2–3 times/month 12.5% once/month
Attack duration [in seconds]	21.7 ± 42.6 (6–180)	10.5 ± 1.7 (3–60)	35.8 ± 60.0 (3–180)
Total number of attacks	437.5 ± 458.5	549.1 ± 578.9	312.0 ± 251.1
Number of attacks in the last 3 months	60.1 ± 47.1	60.0 ± 39.6	60.3 ± 58.0
Time since the first attack [in yrs]	3.4 ± 3.7	3.1 ± 2.9	3.8 ± 4.6
Time since the last attack [in days]	32.8 ± 57.2 (1–210)	40.1 ± 63.0 (1–210)	23.8 ± 51.6 (1–150)
Accompanying symptoms	14/18 (77.8%)	9/10 (90%)	5/8 (62.5%)
Nausea and/or vomiting	4/18 (22.2%)	2/10 (20%)	2/8 (25%)
Perspiration	1/18 (5.6%)	0/10 (0%)	1/8 (12.5%)
Oscillopsia	14/18 (77.8%)	8/10 (80%)	6/8 (75%)
Disturbances of gait and stance	7/18 (38.9%)	5/10 (50%)	2/8 (25%)
Headache	3/18 (16.7%)	1/10 (10%)	2/8 (25%)
History of migraine	2/18 (11.1%)	1/10 (10%)	1/8 (12.5%)
Hypacusis	3/18 (16.7%)	1/10 (10%)	2/8 (25%)
Tinnitus	4/18 (22.2%)	2/10 (20%)	2/8 (25%)
Feeling of pressure within ear	1/18 (5.6%)	1/10 (10%)	0/8 (0%)
Under therapy with an anticonvulsant^a^	17/18 (94.4%)	10/10 (100%)	7/8 (87.5%)
Improvement under therapy	10/18 (83.3%)	10/10 (100%)	25% No improvement 75% Unknown

^a^Carbamazepine (200–600 mg/d), oxcarbazepine (900 mg/d), or lacosamide (100–300 mg/d)

Eleven patients were treated with carbamazepine (5 dVP: minimal dose 200 mg/d, maximal dose 600 mg/d; 6pVP: minimal dose 200 mg/d, maximal dose 800 mg/d), 3 patients were treated with lacosamide (2 dVP, 1 pVP: 100 mg/d) and 2 patients received oxcarbazepine (1 dVP, 1 pVP: 900 mg/day). Two patients received a combination of lacosamide (2 dVP: minimal dose 100 mg/d, maximal dose 300 mg/d) with either carbamazepine (200 mg/d) or oxcarbazepine (900 mg/dl).

### Clinical examination and neurophysiological testing

In 8/18 VP patients (44.4%; dVP: 3/10, 30%; pVP: 5/8, 62.5%), the neurophysiological testing pattern was unremarkable. 7/18 VP patients (38.9%; dVP: 5/10, 50%; pVP: 2/8, 25%) showed a clear pattern of unilateral reduction of audiovestibular function. The remaining 3/18 VP patients (16.7%; dVP: 2/10, 20%; pVP: 1/8, 12.5%) revealed complex neurophysiological patterns representing decreased as well as increased eighth cranial nerve function, that is, a combination of nerve lesion (= hypofunction) and nerve irrigation (= hyperexcitability) within the same side. Beyond that, no significant group differences or correlations with clinical parameters were found. A detailed description of the clinical and neurophysiological results is given in Table 2.

**Table 2:** Clinical and neurophysiological testing

	VP		NP	
	All	Definite	Probable	All
	*n* = 18	*n* = 10	*n* = 8	*n* = 18
Age [in years]	52.6 ± 18.1	52.5 ± 8.8	63.6 ± 8.9	50.3 ± 16.5
Age range	28–84	29–61	49–77	25–78
Gender	6 females	3 females	4 females	13 females
Handedness	17 RH, 1 LH	10 RH	7 RH, 1LH	18 RH
Clinical pattern	44.4% Unremarkable 38.9% Lesion 16.7% Lesion & Irrigation	30% Unremarkable 50% Lesion 20% Lesion & Irrigation	62.5% Unremarkable 25% Lesion 12.5% Lesion & Irrigation	100% Unremarkable – –
TN	1/18 (5.6%)	1/10 (10%)	0/8 (0%)	0/18 (0%)
SPN	1/18 (5.6%)	1/10 (10%)	0/8 (0%)	0/18 (0%)
Nystagmus after hyperventilation	5/14 (35.7%)	3/8 (37.5%)	2/6 (33.3%)	0/18 (0%)
Ocular torsion	0/18 (0%)	0/10 (0%)	0/8 (0%)	0/18 (0%)
SVV deviation	3/18 (16.7%)	2/10 (20%)	1/8 (12.5%)	0/18 (0%)
HIT pathological	6/18 (33.3%)	5/10 (50%)	1/8 (12.5%)	0/13 (0%)
HIT AI [%]	7.2 ± 6.1 (0.5–24.4)	9.0 ± 7.5 (0.5–24.4)	5.0 ± 2.7 (1.1–10.8)	5.2 ± 5.3 (0- 17.4)
Calorics pathological	4/18 (22.2%)	4/10 (40%)	0/8 (0%)	0/13 (0%)
Calorics AI [%]	23.2 ± 25.3 (4.0–88.7)	26.1 ± 23.2 (4.8–80.4)	19.7 ± 28.9 (4.0–88.7)	13.4 ± 10.3 (2.0–36.2)
cVEMP pathological	3/11 (27.3%)	3/6 (50%)	0/5 (0%)	0/7 (0%)
cVEMP amplitude AI [%]	19.4 ± 14.6 (3.0–52.0)	21.8 ± 18.6 (3.0–52.0)	17.0 ± 11.0 (4.0–31.0)	14.3 ± 13.9 (0.2–37.3)
oVEMP pathological	3/11 (27.3%)	3/6 (50%)	0/5 (0%)	0/7 (0%)
oVEMP amplitude AI [%]	17.0 ± 10.9 (2.0–36.0)	13.2 ± 7.9 (5.0–25.0)	20.8 ± 13.1 (2.0–36.0)	8.3 ± 7.6 (2.1–21.3)
PTA pathological	5/16 (31.3%)	3/9 (33.3%)	2/7 (28.6%)	2/3 (60%)
PTA presbycusis-typical	3/16 (18.8%)	0/9 (0%)	3/7 (42.9%)	2/2 (100%)
AEP	2/5 (40%)	1/2 (50%)	1/3 (33.3%)	0/0 (0%)

Abbreviations: *AEP* auditory evoked potential, *AI* asymmetry index, *cVEMP* cervical VEMP, *HIT* head-impulse test, *LH* left-handed, *oVEMP* ocular VEMP, *PTA* pure tone audiometry, *RH* right-handed, *SPN* spontaneous nystagmus, *SVV* subjective visual vertical, *TN* triggered nystagmus, *VEMP* vestibular evoked myogenic potential

### MR imaging approach tailored to VP

#### Neurovascular compression (NVC) and nerve angulation (NA)* (aim i)*

In 15/18 VP patients (83.3%; dVP: 8/10, 80%; pVP: 7/8, 87.5%) and 10/18 NP (55.6%), a NVC between the eighth cranial nerve and a blood vessel was detected. The contacts were documented between the nerve and the anterior inferior cerebellar artery (AICA; VP: 10/15, NP: 10/10), the posterior inferior cerebellar artery (PICA; 2/15 VP), the superior cerebellar artery (SCA; 1/15 VP), and the vertebral artery (VA; 1/15 VP). In another patient, the NVC was between a vein and the nerve. The VP mean distance between the PE and the NVC was 6.1 ± 2.8 mm (range 1.5–9.6 mm; dVP 6.2 ± 3.1 mm, range 1.8–9.6 mm; pVP 6.0 ± 2.7 mm, range 1.5–9.5 mm), the mean VP diameter of the compressing vessels was 0.7 ± 0.1 mm (dVP 0.7 ± 0.1 mm, range 0.6–0.8 mm; pVP 0.8 ± 0.1 mm, range 0.6–1.0 mm). The NP mean distance between PE and NVC was 6.6 ± 4.3 mm (range 1.3–14.2 mm), between NVC and IAC 9.7 ± 1.3 mm (range 7.8–11.9 mm), and the mean NP diameter 0.8 ± 0.3 mm (range 0.5–1.3 mm). NA at the contact site was seen in 8/15 NVC (53.3%; dVP: 6/8, 75%; pVP: 2/7, 28.5%). In comparison, NA was only seen in 2/10 NVC (20%) in NP.

In 6/18 VP patients (33.3%; dVP: 1/10, 10%; pVP: 5/8, 62.5%), the neurophysiological testing pattern and course of the nerve were unremarkable. Clinical side and NVC were found to correspond in 3/18 VP (16.7%, dVP 3/10, 30%), contradictory in 3/18 VP (16.7%; dVP 2/10, 20%, pVP 1/8, 12.5%), or inconclusive in 6/18 VP (33.3%; dVP 4/10, 40%, pVP 2/8, 25%). A detailed description of the NVC results can be seen in Table 3.

**Table 3:** Neurovascular compression (NVC) and nerve angulation (NA) results

	VP		NP	
	All	Definite	Probable	All
NVC incidence	15/18 (83.3%)	8/10 (80%)	7/8 (87.5%)	10/18 (55.6%)
NVC location				
Cisternal	16/20 (75%)	7/10 (70%)	9/10 (90%)	10/16 (62.5%)
Meatal	4/20 (25%)	3/10 (30%)	1/10 (10%)	6/16 (37.5%)
NVC side				
Unilateral	10/18 (55.6%)	6/10 (60%)	4/8 (50%)	2/10 (20%)
Bilateral	5/18 (27.8%)	2/10 (20%)	3/8 (37.5%)	7/10 (70%)
To clinical side				
Norm	6/18 (33.3%)	1/10 (10%)	5/8 (62.5%)	18/18 (100%)
Corresponding	3/18 (16.7%)	3/10 (30%)	–	–
Contradictory	3/18 (16.7%)	2/10 (20%)	1/8 (12.5%)	–
Inconclusive	6/18 (33.3%)	4/10 (40%)	2/8 (25%)	–
Vessel				
AICA	10/15 (66.7%)	5/8 (62.5%)	5/7 (71.4%)	10/10 (100%)
PICA	2/15 (13.3%)	2/8 (25%)	–	–
SCA	1/15 (6.7%)	1/8 (12.5%)	–	–
VA	1/15 (6.7%)	–	1/7 (14.3%)	–
Venous	1/15 (6.7%)	–	1/7 (14.3%)	–
NA	8/15 (53.3%)	6/8 (60%)	2/7 (28.6%)	2/10 (20%)
PE to NVC [mm]	6.1 ± 2.8 (1.5–9.6)	6.2 ± 3.1 (1.8–9.6)	6.0 ± 2.7 (1.5–9.5)	6.6 ± 4.3 (1.3–14.2)
PE to IAC [mm]	10.0 ± 1.5 (7.5–13.1)	9.9 ± 2.0 (7.5–13.1)	10.1 ± 1.0 (8.8–11.3)	9.7 ± 1.3 (7.8–11.9)
Diameter [mm]	0.7 ± 0.1 (0.6–1.0)	0.7 ± 0.1 (0.6–0.8)	0.8 ± 0.1 (0.6–1.0)	0.8 ± 0.3 (0.5–1.3)

Abbreviations: *AICA* anterior inferior cerebellar artery, *IAC* internal auditory canal, *NA* nerve angulation/distortion, *NVC* neurovascular compression, *PE* point of entry/exit for afferent/efferent fibers out of/into the brainstem, *PICA* posterior inferior cerebellar artery, *SCA* superior cerebellar artery, *VA* vertebral artery

#### DTI tractography of the vestibular nerve* (aim ii, aim iv)*

**Fig. 2:**
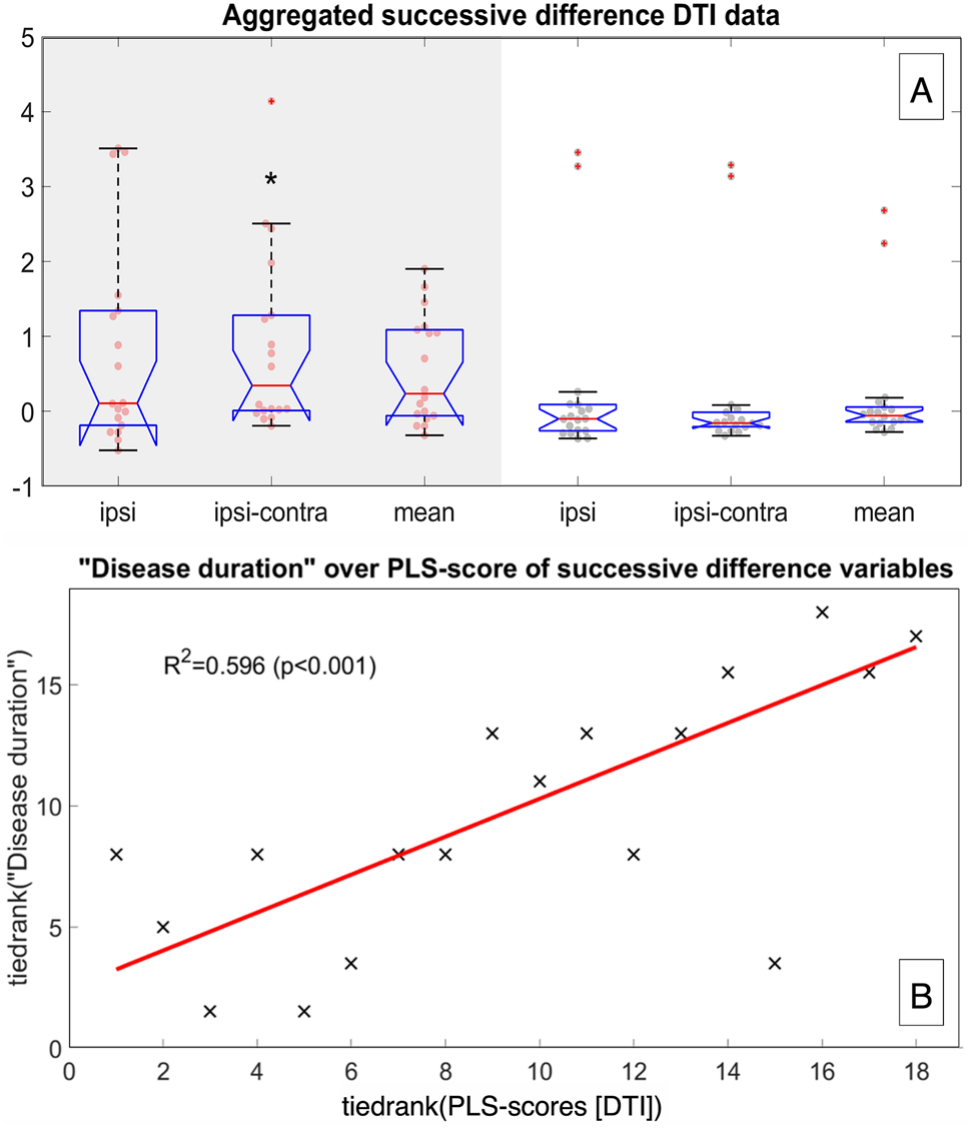
DTI tractography results. Compiled results of DTI group differences (**A**) and correlations (**B**): The upper graphic (**A**) shows the aggregated group difference data for the ipsilateral, the difference between ipsilateral and contralateral sides, and the average (mean) of both sides as boxplots with overlaid data points. The VP data is shown in red with a gray background, and the NP data is shown in black with a white background. Significant differences in the median between the groups are marked by a black star “*”, with a significance level *p* < 0.05. The lower graphic (**B**) depicts PLS rank-correlations between disease parameter “disease duration” and the 1st order of successive differences. The scatter plot of the ranked “disease duration” data and ranks of the 1st order successive difference PLS scores are shown as black crosses, with an overlayed trendline in red. The explained variance r-squared was 0.596 with significance *p* < 0.001 (no failures in 1000 permutation tests)

Average (l) and localized (ll) tensor map approaches showed no significant differences or correlations. However, within the successive difference map approach (lll), the most significant group differences (VP vs. NP) were found for the 1st order successive difference aggregated data collections (1st principal component). This means that in the overall effect along the whole nerve (between PE and IAC), VP showed a higher difference between the ipsilateral and contralateral nerve than NP, where both sides were similar. This supports the theory that there is a measurable structural difference in the affected nerve compared to the non-affected nerve in VP. An overview can be seen in Table 4. Here, VP and NP differed significantly in their DTI parameter median difference (Δ in FA, RD, AD, MD) between the ipsilateral and contralateral side of the 1st order successive difference data (MSSD, *p* < 0.05, corrected for multiple testing). The aggregated data for the ipsilateral and the difference between the ipsilateral vs. contralateral side and the average (mean) are shown in Fig. 2A.

**Table 4:** Structural quantification of the eighth cranial nerve

DTI parameters	VP		NP	
	All	Definite	Probable	All
Fractional anisotropy (FA)				
Ipsilateral	0.1592 ± 0.0385	0.1504 ± 0.0167	0.1701 ± 0.0548	
Mean	0.1604 ± 0.0230	0.1565 ± 0.009	0.1652 ± 0.0336	0.1528 ± 0.0299
Δ	− 0.0024 ± 0.0392	− 0.0121 ± 0.0242	0.0098 ± 0.0518	0.0018 ± 0.0260
FA MSSD				
Ipsilateral	1.908*e − *3 ± 2.862*e − *3	2.473*e − *3 ± 3.757*e − *3	1.212*e − *3 ± 0.847*e − *3	
Mean	2.674*e − *3 ± 3.572*e − *3	2.735*e − *3 ± 3.37*e − *3	2.598*e − *3 ± 4.046*e − *3	0.855*e − *3 ± 0.454*e − *3
Δ	− 1.532*e − *3 ± 6.679*e − *3*✦*	− 0.52*e − *3 ± 6.893*e − *3	− 2.793*e − *3 ± 6.632*e − *3	0.656*e − *3 ± 0.931*e − *3*✦*
Radial diffusivity (RD)				
Ipsilateral	2.3997 ± 0.4198	2.4275 ± 0.3895	2.3649 ± 0.4800	
Mean	2.4064 ± 0.3451	2.4486 ± 0.3103	2.3536 ± 0.3996	2.2799 ± 0.2675
Δ	− 0.0134 ± 0.3857	− 0.0422 ± 0.3677	0.0226 ± 0.4298	0.0952 ± 0.2742
RD MSSD				
Ipsilateral	9.691*e − *4 ± 1.397*e − *3	4.734*e − *4 ± 5.624*e − *4	1.589*e − *3 ± 1.882*e − *3	
Mean	2.541*e − *3 ± 6.488*e − *3	3.991*e − *4 ± 3.512*e − *4	5.219*e − *3 ± 9.345*e − *3	8.471*e − *4 ± 1.930*e − *3
Δ	− 3.145*e − *3 ± 1.104*e − *2*✦*	1.486*e − *4 ± 5.045*e − *4	− 7.261*e − *3 ± 1.615*e − *2	3.975*e − *4 ± 3.244*e − *3*✦*
Axial diffusivity (AD)				
Ipsilateral	3.022 ± 0.4824	3.045 ± 0.4975	2.993 ± 0.4953	
Mean	3.0475 ± 0.3979	3.099 ± 0.3898	2.983 ± 0.4250	2.869 ± 0.3001
Δ	− 0.0509 ± 0.4389	− 0.108 ± 0.4166	0.020 ± 0.4840	0.124 ± 0.3851
AD MSSD				
Ipsilateral	8.618*e − *4 ± 1.301*e − *3	4.294*e − *4 ± 4.750*e − *4	1.402*e − *3 ± 1.793*e − *3	
Mean	2.606*e − *3 ± 6.760*e − *3	4.142*e − *4 ± 3.672*e − *4	5.346*e − *3 ± 9.765*e − *3	8.412*e − *4 ± 1.902*e − *3
Δ	− 3.488*e *− 3 ± 1.154*e *− 2*✦*	3.048*e *− 5 ± 5.120*e *− 4	− 7.886*e *− 3 ± 1.683* e *− 2	3.664* e *− 4 ± 3.293*e *− 3*✦*
Mean diffusivity (MD)				
Ipsilateral	2.607 ± 0.437	2.633 ± 0.424	2.574 ± 0.481	
Mean	2.620 ± 0.362	2.666 ± 0.336	2.563 ± 0.407	2.476 ± 0.273
Δ	− 0.026 ± 0.399	− 0.064 ± 0.381	0.022 ± 0.441	0.105 ± 0.309
MD MSSD				
Ipsilateral	9.191*e − *4 ± 1.359*e − *3	4.493*e − *4 ± 5.279*e − *4	1.506*e − *3 ± 1.848*e − *3	
Mean	2.553*e − *3 ± 6.586*e − *3	3.950*e − *4 ± 3.447*e − *4	5.251*e − *3 ± 9.497*e − *3	8.379*e − *4 ± 1.920*e − *3
Δ	− 3.268*e − *3 ± 1.121*e − *2*✦*	1.085*e − *4 ± 4.937*e − *4	− 7.489*e − *3 ± 1.639*e − *2	0.385*e − *3 ± 3.263*e − *3*✦*
Successive differences SSP (1st PCA)				
Ipsilateral	0.8098 ± 1.3694	0.3259 ± 0.7166	1.4147 ± 1.7724	0.2681 ± 1.1389
Mean	0.5392 ± 0.7161	0.3397 ± 0.5816	0.7887 ± 0.8261	0.2117 ± 0.8312
Δ	0.8684 ± 1.2049*✦*	0.4044 ± 0.5756	1.4485 ± 1.5517	0.2214 ± 1.0932*✦*

*✦*Wilcoxon rank sum test in their medians for VP (all) vs. NP, where *p* < 0.05, or *✦✦* if *p* < 0.001

Abbreviations: Δ = difference between ipsilateral and contralateral side, *AD* axial diffusivity, *FA* fractional anisotropy, *MD* mean diffusivity, *MSSD* Mean of the squared differences of the first (1st) order, *PCA* principal component analysis, *RD* radial diffusivity, *SSP* statistical summary parameter

PLS correlations between the disease parameters “disease duration” and “total number of attacks” and the 1st order of successive differences were significant at *p* < 0.001 (no failures in 1000 permutation tests) with an r-squared of 0.596. The scatter plot of the ranked “disease duration” data and ranks of the 1st order successive difference PLS scores with an overlayed trendline can be viewed in Fig. 2B.

#### Endolymphatic space (ELS) characterization* (aim iii)*

Following a 3-point ordinal scale [57], semi-quantitative ELS (sqELS) classification showed no evidence of endolymphatic hydrops (ELH) in either VP or NP. However, in a 4-point ordinal scale [49], a mild ELH (grade 1) was discernible in 11/18 patients (VP 61.1%, dVP 5/10, 50%; pVP 6/8, 75%). This means that the extent of ELH was low. ELH was most pronounced on the ipsilateral side and in definite VP patients (VP: $sq{ELS}_{ipsi}^{VP}$: grade 0.4 $\pm$ 0.6, range 0–1.5; dVP: $sq{ELS}_{ipsi}^{dVP}$: grade 0.5 $\pm$ 0.6, range 0–1.5; pVP:$sq{ELS}_{ipsi}^{pVP}$: grade 0.4 $\pm$ 0.6, range 0–1.5). This pattern was mirrored in the volumetric ELS (vELS) quantification (VP: $v{ELS}_{ipsi}^{VP}$: 9.6 $\pm$ 4.2 mm^3^, range 4.7–17.9 mm^3^; dVP: $v{ELS}_{ipsi}^{dVP}$: 10.2 $\pm$ 4.6 mm^3^, range 5.2–17.9 mm^3^; pVP:$v{ELS}_{ipsi}^{pVP}$: 8.7 $\pm$ 3.7 mm^3^, range 4.7–15.4 mm^3^). In comparison, 5/18 NP (27.8%) showed a mild ELH with an altogether lower mean semiquantitative and volumetric ELS quantification ($sq{ELS}_{mean}^{NP}$: grade 0.2 $\pm$ 0.4, range 0–1; $v{ELS}_{mean}^{NP}$: 8.9 $\pm$ 2.5 mm^3^, range 5.2–13.9 mm^3^). VP and NP differed significantly regarding the extent of the asymmetry index of the inner ear and vestibulum volume (*p* < 0.05, ANOVA, corrected for multiple testing). Definite VP and NP, on the other hand, differed significantly regarding the extent of the asymmetry index of the vestibulum volume and difference in volume between ipsi- and contralateral side (*p* < 0.05, ANOVA, corrected for multiple testing). No correlations were found between electrophysiological data or degree of ELH and disease duration or number of attacks. A detailed description of the ELS results can be seen in Table 5.

**Table 5:** Semi- and 3D-quantification of the endolymphatic space

	VP		NP	
	All	Definite	Probable	All
To clinical side				
Norm	2/18 (11.1%)	1/10 (10%)	1/8 (12.5%)	18/18 (100%)
Corresponding	3/18 (16.7%)	1/10 (10%)	2/8 (25%)	–
Inconclusive	13/18 (72.2%)	8/10 (80%)	5/8 (62.5%)	–
To NVC side				
Norm	1/18 (5.6%)	1/10 (10%)	–	16/18 (88.8%)
Corresponding	2/18 (11.1%)	–	2/8 (25%)	1/18 (11.1%)
Contradictory	3/18 (16.7%)	2/10 (20%)	1/8 (12.5%)	1/18 (11.1%)
Inconclusive	12/18 (66.6%)	7/10 (70%)	5/8 (62.5%)	–
*Inner ear*				
ELH	11/18 (61.1%)	5/10 (50%)	6/8 (75%)	5/18 (27.8%)
Side of ELH				
Unilateral	7/18 (38.9%)	3/10 (30%)	4/8 (50%)	3/18 (16.7%)
Bilateral	4/18 (22.2%)	2/10 (20%)	2/8 (25%)	2/18 (11.1%)
*sq*ELS [grade]				
Ipsilateral	0.4 ± 0.6 (0–1.5)	0.5 ± 0.6 (0–1.5)	0.4 ± 0.4 (0–1)	0.2 ± 0.4 (0–1)
Contralateral	0.3 ± 0.4 (0–1)	0.3 ± 0.4 (0–1)	0.3 ± 0.4 (0–1)	0.2 ± 0.3 (0–1)
AI [%]	11.1 ± 79.8 (− 1–100)	16.7 ± 75.3 (− 100–100)	5.6 ± 90.5 (− 100–100)	0 ± 81.7 (− 100–100)
*v*ELS [mm^3^]				
Ipsilateral	9.6 ± 4.2 (4.7–17.9)	10.2 ± 4.6 (5.2–17.9)	8.7 ± 3.7 (4.7–15.4)	8.9 ± 2.5 (5.2–13.9)
Contralateral	7.9 ± 2.9 (4.7–13.0)	8.5 ± 3.4 (4.2–13.0)	7.1 ± 2.2 (4.6–10.6)	
Δ	1.7 ± 2.2 (− 2.4–6.1)	1.7 ± 2.2 (− 0.6–6.1)	1.7 ± 2.4 (− 2.4–4.8)	0.1 ± 1.9 (− 2.6–3.9)
AI [%]	8.6 ± 11.3 (− 20.4–25.9)*✦*	8.3 ± 9.1 (− 4.3–20.8)	8.8 ± 14.3 (− 20.4–25.9)	0.1 ± 10.8 (− 20.3–16.4)*✦*
TFS [mm^3^]	275.8 ± 26.3 (232.1–321.7)	277.5 ± 24.3 (247.6–311.3)	273.8 ± 30.3 (232.1–321.7)	261.9 ± 34.3 (193.4–309.3)
*Cochlea*				
ELH	6/18 (33.3%)	3/10 (30%)	3/8 (37.5%)	2/18 (11.1%)
Side of ELH				
Unilateral	4/18 (22.2%)	2/10 (20%)	2/8 (25%)	1/18 (5.6%)
Bilateral	2/18 (11.1%)	1/20 (10%)	1/8 (12.5%)	1/18 (5.6%)
ELS [grade]				
Ipsilateral	0.4 ± 0.6 (0–2)	0.5 ± 0.7 (0–2)	0.4 ± 0.5 (0–1)	0.2 ± 0.4 (0–1)
Contralateral	0.2 ± 0.4 (0–1)	0.1 ± 0.3 (0–1)	0.3 ± 0.5 (0–1)	0.1 ± 0.3 (0–1)
AI [%]	50 ± 75.6 (− 100–100)	75 ± 50 (0–100)	25 ± 95.7 (− 100–100)	25 ± 95.7 (− 100–100)
ELS [mm^3^]				
Ipsilateral	2.9 ± 1.5 (1.1–6.5)	3.1 ± 1.8 (1.1–6.5)	2.7 ± 1.2 (1.3–4.4)	2.5 ± 1.2 (0.5–5.2)
Contralateral	2.6 ± 1.3 (1.0–5.6)	2.8 ± 1.3 (1.2–3.4)	2.4 ± 1.2 (1.0–3.9)	
Δ	0.3 ± 1.2 (− 1.6–3.2)	0.3 ± 1.4 (− 1.5–3.2)	0.4 ± 2.0 (− 2.3–4.5)	0.1 ± 1.4 (− 2.7–2.7)
AI [%]	4.9 ± 21.9 (− 40.8–36.7)	2.2 ± 26.6 (− 40.8–36.4)	8.2 ± 15.5 (− 13.2–36.7)	5.8 ± 32.7 (− 71.4–81.8)
TFS [mm^3^]	90.8 ± 9.3 (73.1–104.68)	90.3 ± 9.0 (78.8–104.7)	91.4 ± 10.3 (73.1–104.7)	87.3 ± 15.0 (51.1–120.5)
*Vestibulum*				
ELH	9/18 (50%)	4/10 (40%)	5/8 (62.5%)	5/18 (27.8%)
Side of ELH				
Unilateral	6/18 (33.3%)	2/10 (20%)	4/8 (50%)	3/18 (16.7%)
Bilateral	3/18 (16.7%)	2/20 (20%)	1/8 (12.5%)	2/18 (11.1%)
ELS [grade]				
Ipsilateral	0.4 ± 0.5 (0–1)	0.4 ± 0.5 (0–1)	0.4 ± 0.5 (0–1)	0.2 ± 0.4 (0–1)
Contralateral	0.4 ± 0.5 (0–1)	0.4 ± 0.5 (0–1)	0.4 ± 0.5 (0–1)	0.2 ± 0.4 (0–1)
AI [%]	0 ± 89.4 (− 100–100)	0 ± 89.4 (− 100–100)	0 ± 100 (− 100–100)	0 ± 89.4 (− 100–100)
ELS [mm^3^]				
Ipsilateral	6.7 ± 3.1 (1.6–12.0)	7.1 ± 3.3 (2.9–12.0)	6.1 ± 3.0 (1.6–11.5)	6.6 ± 2.2 (2.9–11.9)
Contralateral	5.3 ± 2.0 (3.0–9.2)	5.7 ± 2.3 (3.0–9.2)	4.8 ± 1.3 (3.5–7.0)	
Δ	1.4 ± 1.6 (− 2.3–4.4)	1.4 ± 1.3 (− 0.6–3.6)*✧*	1.4 ± 2.0 (− 2.3–4.4)	− 0.2 ± 1.7 (− 3.7–2.1)*✧*
AI [%]	8.6 ± 15.6 (− 41.1–28.5)*✦*	9.8 ± 9.0 (− 6.4–19.5)*✧*	7.3 ± 21.3 (-41.1–28.5)	− 1.4 ± 14.2 (− 32.5–25.8)*✦,✧*
TFS [mm^3^]	185.0 ± 19.1 (150.3–218.4)	187.1 ± 17.4 (168.1–211.0)	182.4 ± 22.1 (150.3–218.4)	175.8 ± 19.6 (144.4–208.5)

*✦ ANOVA for VP (all) vs. NP, where p* < *0.05, or ✦✦ if p* < *0.001*

*✧ ANOVA for VP (definite) vs. NP, where p* < *0.05, or ✧✧ if p* < *0.001*

Abbreviations: Δ = difference between ipsilateral and contralateral side, *AI* asymmetry index, *ELH* endolymphatic hydrops, *ELS* endolymphatic space, *sq* semi-quantitative, *v* volumetric, *TFS* total fluid space

Clinical side and ELS quantification lateralization were inconclusive in 13/18 VP (72.2%; dVP 8/10, 80%; pVP 5/8, 62.5%), and found to correspond in 3/18 VP (16.7%, dVP 1/10, 10%; pVP 2/8, 25%). In 2/18 VP patients (11.1%; dVP 1/10, 10%; pVP 1/8, 12.5%), the neurophysiological testing pattern and ELS were unremarkable. NVC side and ELS quantification lateralization were inconclusive in 12/18 VP (66.6%; dVP 7/10, 70%, pVP 5/8, 62.5%), contradictory in 3/18 VP (16.7%; dVP 2/10, 20%; pVP 1/8, 12.5%), found to correspond in 2/18 VP (11.1%; pVP 2/8, 25%), or normal in 1/18 (5.6%; dVP 1/10, 10%).

### Interparametric correlations (*aim iv*)

**Fig. 3:**
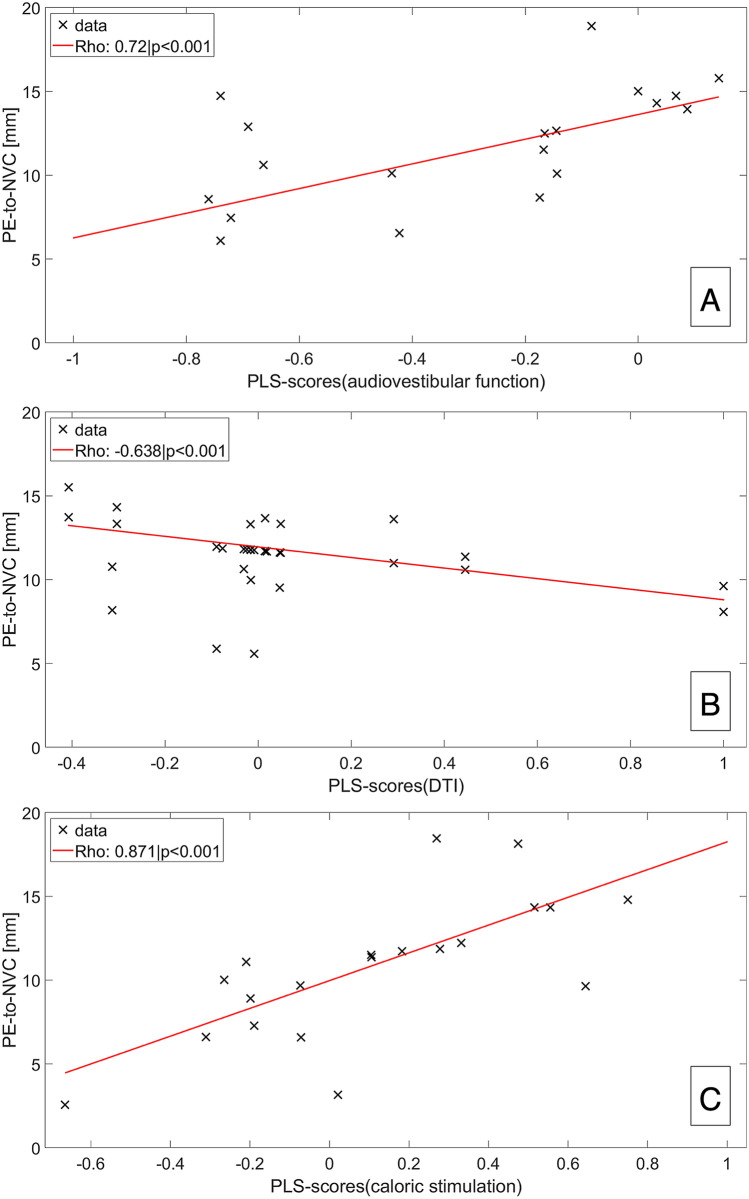
Interparametric correlations. Scatter plot depiction with trendlines of the correlations of “distance between PE and NVC (PE-to-NVC [mm])” and the eighth cranial nerve (**A**) audiovestibular function, i.e. neurophysiological testing (caloric stimulation, HIT and PTA), (**B**) structural nerve integrity (DTI SSPs, or deviations of successive DTI differences), and inner ear ELS volumes (**C**). A decreasing distance between PE to NVC can be translated into the transition zone of the eighth nerve (6-15 mm as measured from the PE, where oligodendrocytes change into Schwann cells [89]), and the root-entry zone from 6 mm downwards [88]. In consequence, the positive correlation of PE-to-NVC to audiovestibular function (**A**, Roh 0.72, *p* < 0.001) indicates better function the more the NVC lies outside the root-entry area of the intracisternal part of the eighth cranial nerve, and not covered by central myelin (oligodendrocytes), but peripheral myelin (Schwann cells). Conversely, the negative correlation of PE-to-NVC to nerval structural microstructure DTI SSPs (**B**, Roh − 6.638, *p* < 0.001), or inner ear ELS volumes (**C**, Roh − 6.604, *p* < 0.001) indicates less nerve deterioration, or less ELS volume the higher the PE-to-NVC distance. Altogether, these correlations link the suttle clinical, diagnostic, DTI-proxy for nerve microstructure, and ELS volume findings in VP to NVC, and in particular to NVC being in the root-entry zone. Abbreviations: *ELS* endolymphatic space, *NVC* neurovascular compression, *PE* point of entry/exit for afferent/efferent fibers out of/into the brainstem, *PE-to-NVC* distance between PE and NVC [mm], *SSP* summary statistic parameters, or deviations in successive differences.

The distance between PE and NVC (PE-to-NVC [mm]) correlated with audiovestibular function, i.e., neurophysiological testing (Roh 0.72, *p* < 0.001), structural nerve integrity, i.e., DTI SSPs (Roh − 6.638, p < 0.001), and inner ear ELS (Roh − 6.604, *p* < 0.001). Scatter plots with trendlines are shown in Fig. 3.

## Discussion

The current study focused on a detailed characterization of structures affecting the cisternal part of the eighth cranial nerve to increase the diagnostic value of MRI in patients with vestibular paroxysmia (VP) using an MR imaging approach tailored to VP. The high-resolution MRI sequences were used to determine neurovascular compression (NVC) and nerve angulation/distortion (NA), structural nerve integrity via diffusion tensor imaging (DTI), and in-vivo non-invasive verification of endolymphatic hydrops (ELH). The findings were the following: 1. Previously established evidence on the typical clinical and heterogenous diagnostic picture could be confirmed. 2. Nerve angulation (NA) increases NVC diagnostic specificity in VP *(aim i)*; 3. DTI showed structural differences in the affected vestibular nerve in VP at group-level (*p* < 0.05), *(aim ii)*; 4. Endolymphatic space (ELS) asymmetry index was mildly but significantly increased at group-level (*p* < 0.05), *(aim iii)*; 5. “Disease duration” and “total number of attacks” correlated positively with DTI, i.e., with structural nerve integrity (Roh = 0.594, *p* < 0.001), *(aim iv)*; 6. Distance between PE (point of entry/exit for afferent/efferent fibers out of/into the brainstem) and NVC (“PE-to-NVC”) correlated positively with audiovestibular function (Roh = − 0.72, *p* < 0.001) and negatively with structural nerve integrity (Roh = − 0.638, *p* < 0.001), or volume of the inner ear ELS (Roh = − 0.604, *p* < 0.001), *(aim iv).* At the group level, there were significant differences between VP participants and NP. In sum, the predictive value of MRI is increased. However, on a single-subject level, the methodology’s specificity is insufficient to diagnose VP.

In the following, we discuss these findings compared to previously published VP studies and the significance of dedicated VP MRI to quantify ELS and vestibular nerve structural integrity, its clinical implications, and recommendations for future studies and methodical limitations.

### Confirmation of previously established VP evidence (*aim i*)

VP pathophysiology seems to be best explained by a “dual”, i.e., combined peripheral and central pathology [10]. While NVC is a necessary factor for the development of clinical symptoms [58–60], a central cranial nerve nuclei pathology with increased excitability or even a dysfunction at the level of thalamic-cortical projections, as well as on a cortical level with a reduced inhibitory projection to the cranial nerve nuclei develop [61, 62]. Furthermore, additional unknown factors are thought to play a role [63].

This study’s clinical and diagnostic results mirror the clinical picture of this vestibular NVC syndrome. The current VP cohort was characterized by the typical monomorphic clinical syndrome [2–4] and accompanied by a heterogeneous [12, 64] diagnostic neurophysiological picture (25–50% unremarkable, 33–45% pattern of reduction of audiovestibular function [6, 12], 17–30% complex neurophysiological pattern representing decreased as well as increased nerve function [8, 9, 11] as a combination of nerve lesion and nerve irrigation within the same side [10]). NVC was found in 83.3% of the current study and occurred along the whole intracisternal part of the nerve, particularly within the root entry and transition zone. Meatal NVC is rarer but did also occur. Most frequently, the compressing vessel could be attributed to the AICA (75%), but other vessels, such as the vertebral artery (VA, 10%), PICA (5%), or a vein (10%), are possible [10]. This was also true in the current study (AICA 66.7%, PICA 13.3%, VA 6.7%, SCA 6.7%, vein 6.7%). However, vascular loops as normal variants without clinical symptoms were found in about 25–30% (and in our control group about 50%) [65]. As an additional feature, NA increased NVC's specificity in VP [14]. Fittingly, they were 3:1 more frequent in VP than in NP and 2:1 more frequent in definite VP than in probable VP of our study.

### Structural abnormalities of the vestibular nerve revealed by DTI (*aim ii*)

NVC syndromes entail a local peripheral nerve compression. It, therefore, seems reasonable to assume a DTI structural correlation in the affected nerve of VP patients. The current study found measurable DTI structural differences in the median difference between the affected and non-affected side of the first order successive difference data of the eighth cranial nerve in VP at group level (*p* < 0.05; Fig. 2A) that correlated with “disease duration” and “total number of attacks” (*p* < 0.001; Fig. 2B). Nevertheless, the average and localized tensor map approaches were not successful. Different factors could play a role here. One reason may be the low sensitivity of the method. In the future, this could be resolved by choosing high spatial resolution nerve-specific DTI over whole-brain DTI [66], specialized vestibular nerve DWI acquisition strategies for each subsegment [67], and optimized protocols concerning resolution, phase encoding [68], or the number of b-shells [69]. Another reason may be that although local compression of the peripheral nerve by the peripheral NVC is necessary for developing the clinical symptoms, the microstructural lesion does not remain local or is less pronounced than expected. A 7 Tesla T1-sequence with and without contrast agent in 6 VP patients showed no structural abnormalities [13]. Beyond that, to the best of our knowledge, nothing has been published on structural nerve abnormalities in VP.

In contrast, many studies exist on MR structural nerve abnormalities in the trigeminal NVC syndrome, trigeminal neuralgia (TiN), a disease with similar underlying pathophysiology [70]. The results were inhomogeneous, too. Some DTI studies showed microstructural alterations in fractional anisotropy (FA) and mean diffusivity (MD) values between affected and non-affected TiN sides [71–73] that could be used for postoperative longitudinal control [74]. However, other studies showed no correlation between DTI parameters and clinical symptoms, such as illness duration or severity of compression [75].

### Mild ELH in VP discernible (*aim iii*)

Following the 4-point ordinal scale, semi-quantitative classification of the ELS for the vestibulum and cochlea [49, 76], volumetric quantification in the current study disclosed a mild yet significantly increased asymmetry index of the inner ear and vestibulum when compared to NP. These findings are overlooked by a part of the 3-point ordinal scale ELH classification of the vestibulum [77]. This can be explained by the fact that these classifications were calibrated on patients with Ménière’s disease when ELH was viewed as pathognomonic to MD, which tends to have a larger ELH [17]. Fittingly and further underlying its pathophysiological relevance, varying degrees of ELH are also nearly universally present in post-mortem human temporal bone [78, 79] and iMRI studies on subjects with a history of MD [16, 18, 19, 32]. However, in-vivo iMRI studies also showed a relatively high prevalence in the healthy population (via iMRI [80], as well as in post-mortem studies [81]) and in patients with other vestibular disorders such as vestibular migraine [18, 19], vestibular schwannoma [82], or bilateral vestibulopathy [83]. Consequently, ELH is being re-evaluated as a non-pathognomonic phenomenon that should not directly indicate MD [84]. In this context, finer graduation in the lower ELH grades is useful not to overlook milder cases of ELH, be it in a 3-point ordinal scale [57] or a 4-point ordinal scale ELH classification of the vestibulum [49].

With respect to the current study, VP seems to be another vestibular disorder that can entail mild ELH. Although nonspecific, this finding may be one of several parameters that help diagnose the affected side (view Fig. 3). ELH has been tied to many pathophysiological theories, such as post-viral autoimmune mechanisms, anatomic or vascular abnormalities affecting endolymph resorption, and different factors relating to water homeostasis [85–87]. The pathogenetic mechanism for higher inner ear ELS volumes in VP seems to be related to the NVC lying in the root entry zone of the intracisternal part of the eighth cranial nerve (*p* < 0.001). The nerve compression might then lead to a disturbance in the inner ear fluid homeostasis, either directly or via a central pathology inducing hyperexcitability within the cranial nerve nuclei or reduced inhibitory projection to the cranial nerve nuclei.

### Interparametric analyses corroborate NVC syndrome (*aim iv*)

The nearer the NVC was to the PE, the poorer audiovestibular function (*p* < 0.001) and nerve structural integrity (*p* < 0.001), and the larger inner ear ELS (*p* < 0.001) were. The intracisternal part of the eighth nerve starts covered by central myelin (i.e., oligodendrocytes, also called root entry zone), then 6–15 mm as measured from the PE [88] oligodendrocytes change into Schwann cells [89], which is called the transition zone. The present interparametric analyses corroborate the pathophysiological theory [2, 10] that the intracisternal part of the eighth cranial nerve covered by central myelin (i.e., oligodendrocytes) seems to be particularly vulnerable for deficits induced by NVC and that the NVC seems to be a necessary precondition factor to develop VP.

### Clinical implications and recommendations for future studies

In VP, no “vestibular” symptom has a precise meaning in topology or nosology [1], which makes corroborating evidence from quantifiable features all the more critical. The current VP study should be understood as a proof-of-concept study for using MR imaging tailored to VP as a complementary clinical tool to investigate the vestibular end organ. On a single-subject level, MRI tailored to VP can deliver additional quantifiable features for diagnosing VP. Especially the verification of NA and mild ELH can increase the overall predictive value of MRI. Nevertheless, the specificity of the methodology is not yet sufficient to diagnose VP by MR imaging.

The improvement of the MR sequences, especially DTI (as mentioned in “Mild ELH in VP discernible, (*aim iii*)”), holds potential. This study’s sequence acquisition parameters represent a compromise between novel technical possibilities, resolution, and reasonable scan time while enabling a wide range of clinical applications. Novel MR neurography imaging techniques might prove helpful for the investigation of the structural integrity of the vestibular nerve, such as high-resolution isotropic [90], driven equilibrium (drive) [91], or 3D cranial nerve [92] MR imaging. In addition, similar to trigeminal nerve analyses, atlas-based segmentation might prove helpful [93]. Another approach to improve the MRI investigation of the neurovascular compression site could be the addition of magnetic resonance subtraction [94]. Furthermore, techniques beyond DTI tractography might unlock the mesoscopic potential of diffusion-weighted imaging [95]. Another aspect would be to investigate the structural and functional connectivity and disturbances in cortical networks since there also seems to be a central aspect in the pathophysiology of VP.

#### Methodological limitations

Two major limitations of the current study need to be considered: First, the limited number of subjects included, and second, the circumstance that *i*MRI imaging for the control group (NP) could only be done in patients with other (neurological) pathologies that did not affect the peripheral or central vestibulocochlear systems. First, given the variation in symptomatology, a higher number of VP participants would be desirable. If possible, 20 participants per clinical pattern should be included. Second, influences of the NP’s underlying neurological pathologies on the ELS appeared unlikely (view inclusion and exclusion criteria, Sect. 2.2.) but cannot entirely be excluded. However, ethical considerations did not allow us to include healthy volunteers without a medical indication for an iMRI with contrast agent. The decision to avoid unnecessary contrast agent application was based on prior findings of signal intensity increases in the dentate nucleus and globus pallidus on T1-weighted MR images after applying MR contrast agents that are still under investigation [96–98]. Third, the study lacks histological confirmation of endolymphatic hydrops since the in-vivo acquisition of histological specimens is currently not possible.

## Conclusion

This study is the first to link eighth cranial nerve function, microstructure, and endolymphatic space changes in VP patients to clinical features and neurovascular compression in the root-entry zone (correlation coefficient > 0.5, *p* < 0.001). Significant differences were reached at group level (ANOVA, *p* < 0.05, corrected for multiple testing). However, moderate specificity of MR NVC and NA evidence, DTI quantification, or ELH verification means they do not suffice to diagnose VP on a single-subject level.

## Abbreviations

3D: Three-dimensional
AD: Axial diffusivity
AEP: Auditory evoked potential
AI: Asymmetry index
AICA: Anterior inferior cerebellar artery
ANOVA: Analysis of variance
Δ: Side difference
DTI: Diffusion tensor imaging
dVP: Definite vestibular paroxysmia
DWI: Diffusion-weighted imaging
ELH: Endolymphatic hydrops
ELS: Endolymphatic space
FA: Fractional anisotropy
FLAIR: Fluid-attenuated inversion recovery
NP: Participants with normal vestibulocochlear testing
HIT: Head-impulse test
IAC: Internal auditory canal
IE-Vnet: Inner ear Vnet
*i*MRI: Delayed intravenous gadolinium-enhanced MRI of the inner ear
LH: Left-handed
MASD: Mean of the absolute differences of the first (1st) order
MA2SD: Mean of the absolute differences of the second (2nd) order
MD: Mean diffusivity
MRI: Magnetic resonance imaging
MSSD: Mean of the squared differences of the 1st order
MS2SD: Mean of the squared differences of the 2nd order
NA: Nerve angulation
NVC: Neurovascular compression
PCA: Principal component analysis
PE: Point of entry/exit for afferent/efferent fibers out of/into the brainstem
PICA: Posterior inferior cerebellar artery
PS: Perilymphatic space
PLS: Projection into latent space
PTA: Pure tone audiometry
pVP: Probable vestibular paroxysmia
RD: Radial diffusivity
RH: Right-handed
SCA: Superior cerebellar artery
SPN: Spontaneous nystagmus
SPACE: Sampling perfection with application-optimized contrasts by using different flip angle evolutions
SSP: Statistical summary parameter
SVV: Subjective visual vertical
TFS: Total fluid space
TN: Triggered nystagmus
TiN: Trigeminal neuralgia
VA: Vertebral artery
VM: Vestibular migraine
VN: Vestibular nerve
VOG: Video-oculography
VP: Vestibular paroxysmia
